# Case Report: bullous pemphigoid during secukinumab treatment for plaque psoriasis: therapeutic challenges and long-term follow-up

**DOI:** 10.3389/fimmu.2026.1964020

**Published:** 2026-10-20

**Authors:** Han Deng, Hongli Rao, Si Jiang, Jun Xie

**Affiliations:** Department of Dermatology, Zhongnan Hospital of Wuhan University, Wuhan, China

**Keywords:** autoimmunity, biologic therapy, bullous pemphigoid, interleukin-17A, psoriasis, secukinumab

## Abstract

**Background:**

Bullous pemphigoid (BP) may occur during interleukin-17A inhibitor therapy in patients with psoriasis. Most reported cases have focused on BP onset and short-term management, with limited information on long-term outcomes and subsequent targeted treatment.

**Case presentation:**

A 68-year-old man with a history of severe plaque psoriasis for more than 20 years developed generalized pruritic tense bullae after 10 injections of secukinumab, despite improvement of his psoriatic lesions. Laboratory testing showed peripheral eosinophilia, elevated total immunoglobulin E, anti-BP180 antibody levels above 400 RU/mL, and an anti-BP230 antibody level of 344.06 RU/mL. Histopathology demonstrated a subepidermal blister with eosinophil-rich inflammation, and direct immunofluorescence showed linear IgG and C3 deposition along the basement membrane zone, confirming BP. Secukinumab was discontinued, and treatment with prednisone, cyclosporine, minocycline, nicotinamide, and topical corticosteroids rapidly controlled blister formation. Psoriasis subsequently remained inadequately controlled despite sequential treatment with tildrakizumab and ustekinumab. In June 2025, BP remained clinically quiescent, although anti-BP180 antibodies exceeded 400 RU/mL, total immunoglobulin E exceeded 1,100 kU/L, and C3 deposition persisted along the basement membrane zone. Deucravacitinib was subsequently administered for approximately 6 months, during which psoriasis improved from moderate to mild severity without clinical recurrence of BP. At the latest structured telephone follow-up, approximately 6 months after deucravacitinib discontinuation, the patient reported that psoriasis remained mild and stable and that no BP manifestations had recurred.

**Conclusion:**

This case provides more than two years of longitudinal follow-up after BP developed during secukinumab treatment. It illustrates the challenges of sequential targeted therapy for coexisting psoriasis and BP and shows that clinical quiescence may coexist with persistent BP-associated serological and immunopathological abnormalities. Continued clinical and immunological monitoring is advisable when switching therapeutic targets in these patients.

## Introduction

Psoriasis and bullous pemphigoid (BP) are distinct immune-mediated dermatoses, yet their coexistence is more frequent than expected. A systematic review and a population-based cohort study demonstrated an increased and bidirectional association between the two diseases (1, 2). Chronic psoriatic inflammation and repeated epidermal injury may expose hemidesmosomal antigens, including BP180 and BP230. Shared IL-17-related inflammation, impaired immune regulation, and keratinocyte- and neutrophil-mediated tissue injury may further contribute to their coexistence, although BP is more strongly characterized by type 2 inflammation, eosinophil activation, autoantibody production, and complement activation (3, 4). This immunological complexity creates a therapeutic challenge because pathway-specific treatment may control one disease while altering the immune response involved in the other (4, 5).

Interleukin-17A (IL-17A) contributes to BP-associated inflammation and has been investigated as a potential therapeutic target (6, 7). At the same time, new-onset or relapsing BP has been reported during treatment with IL-17A inhibitors, including secukinumab (8–10). Most published cases have focused on BP onset and short-term management, with limited information on long-term disease activity or subsequent treatment of psoriasis. We report more than two years of follow-up after BP developed during secukinumab therapy. The patient subsequently received sequential systemic treatment with cyclosporine, tildrakizumab, ustekinumab, and the TYK2 inhibitor deucravacitinib. During follow-up, clinical remission of BP persisted despite elevated anti-BP180 antibodies and continued C3 deposition along the basement membrane zone. This extended clinical course provides insight into the selection of subsequent targeted therapies and the dissociation between clinical and immunological remission in patients with coexisting psoriasis and BP.

## Case description

A 68-year-old man with a more than 20-year history of plaque psoriasis presented to our department in March 2023 because of extensive and poorly controlled skin lesions. Before presentation, his psoriasis had been treated intermittently with topical corticosteroids, oral acitretin, and UVB phototherapy, which provided only partial or temporary improvement. He had not previously received biologic therapy and had no history of pruritic tense bullae or a diagnosis of bullous pemphigoid. Physical examination showed widespread erythematous, infiltrated, and scaly plaques involving approximately 95% of the body surface area, with a Psoriasis Area and Severity Index score of at least 10. Secukinumab was therefore initiated at a dose of 300 mg by subcutaneous injection according to the standard dosing schedule. His psoriatic lesions improved markedly after the first two injections and remained well controlled during the initial treatment period. In October 2023, after a total of 10 secukinumab injections, the patient developed severe pruritus accompanied by numerous erythematous lesions, vesicles, and bullae over the head and neck, trunk, and extremities. At that time, the underlying psoriasis was clinically assessed as moderate. On examination, multiple thick-walled, tense bullae containing clear fluid were observed on erythematous skin, together with erosions, crusts, and residual psoriatic plaques. Nikolsky’s sign was negative, and the oral and genital mucosae were not involved (**Figure 1**).

**Figure 1:**
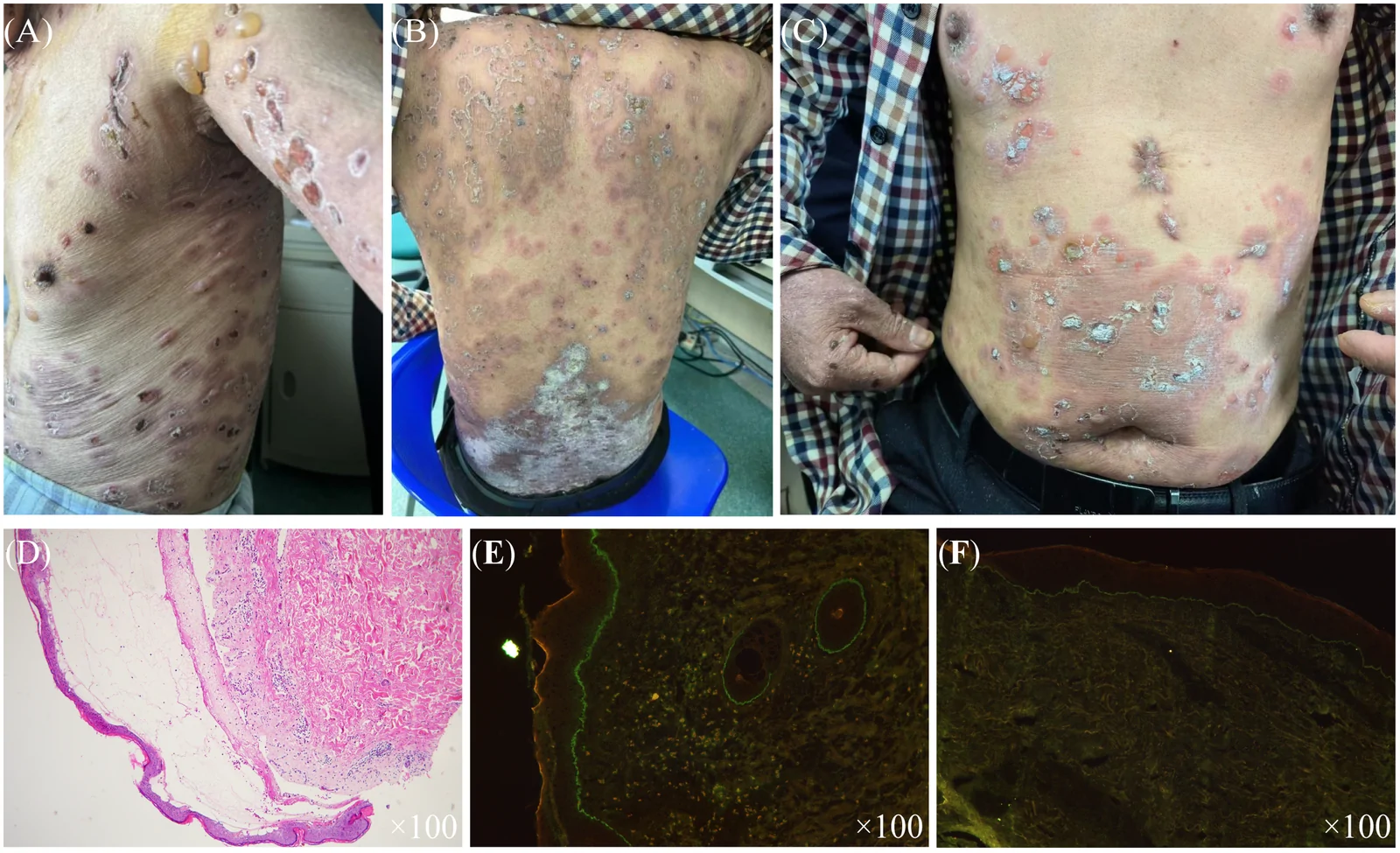
Clinical, histopathological, and immunopathological findings at the onset of bullous pemphigoid during secukinumab treatment. **(A–C)** Widespread psoriatic plaques, erosions, crusts, and multiple tense vesicles and bullae on the trunk and extremities. **(D)** Histopathological examination showing a subepidermal blister with eosinophils and mixed inflammatory cell infiltration in the superficial dermis (hematoxylin and eosin staining; original magnification, ×100). **(E)** Direct immunofluorescence showing linear C3 deposition along the basement membrane zone (original magnification, ×100). **(F)** Direct immunofluorescence showing linear IgG deposition along the basement membrane zone (original magnification, ×100).

## Diagnostic assessment

Laboratory investigations revealed a white blood cell count of 13.80 × 10^9^/L and an absolute neutrophil count of 8.50 × 10^9^/L. Peripheral eosinophilia was present, with eosinophils accounting for 16.4% of leukocytes and an absolute eosinophil count of 2.30 × 10^9^/L. The serum total immunoglobulin E level was elevated to 693 kU/L. Serum anti-BP180 antibody was greater than 400 RU/mL, and anti-BP230 antibody was 344.06 RU/mL. Histopathological examination of a lesional skin biopsy demonstrated a subepidermal cleft and blister formation, with infiltration of eosinophils, neutrophils, and lymphocytes in the superficial dermis. Direct immunofluorescence examination of perilesional skin showed linear deposition of immunoglobulin G and complement component 3 along the basement membrane zone. Based on the clinical, serological, histopathological, and immunopathological findings, bullous pemphigoid (BP) coexisting with plaque psoriasis was diagnosed (**Figure 1**). Given the temporal relationship, a possible association between the clinical onset of BP and secukinumab exposure was considered.

## Therapeutic intervention

Secukinumab was discontinued. The patient was treated with oral prednisone 30 mg/day, cyclosporine 175 mg/day, minocycline 200 mg/day, and nicotinamide 400 mg/day, together with topical corticosteroids and symptomatic antipruritic treatment. No new blisters developed after three days of treatment, and the pruritus gradually subsided. The existing bullae subsequently dried, crusted, and healed. Peripheral eosinophil counts returned to the normal range before discharge. The patient was discharged on prednisone 30 mg/day. After disease control had been achieved, prednisone was tapered by 5 mg every 4 weeks according to the clinical response and was discontinued in April 2024. No new bullae developed during the tapering period. Cyclosporine 175 mg/day was continued until April 2024. BP remained clinically controlled, whereas the psoriatic lesions became increasingly difficult to manage.

## Follow-up and outcomes

Because of persistent psoriasis activity, tildrakizumab was initiated in May 2024. The patient received a second injection one month later, and a transient improvement in the psoriatic lesions was observed at follow-up in July 2024. However, newly developed eczematous lesions subsequently appeared. As a possible treatment-related adverse reaction could not be excluded, tildrakizumab was discontinued in September 2024. Ustekinumab was initiated in January 2025 and continued for approximately five months, but it failed to achieve adequate control of psoriasis.

In June 2025, the patient was readmitted because of persistent plaque psoriasis, which was clinically assessed as moderate at admission. Physical examination showed symmetrically distributed dark-red, scaly plaques on the trunk and extremities, particularly on the lower limbs. Some lesions had coalesced into larger plaques, and erythema and erosions were present on both lower legs. However, no typical tense vesicles or bullae were observed, and BP was considered to be in clinical remission (**Figure 2**).

**Figure 2:**
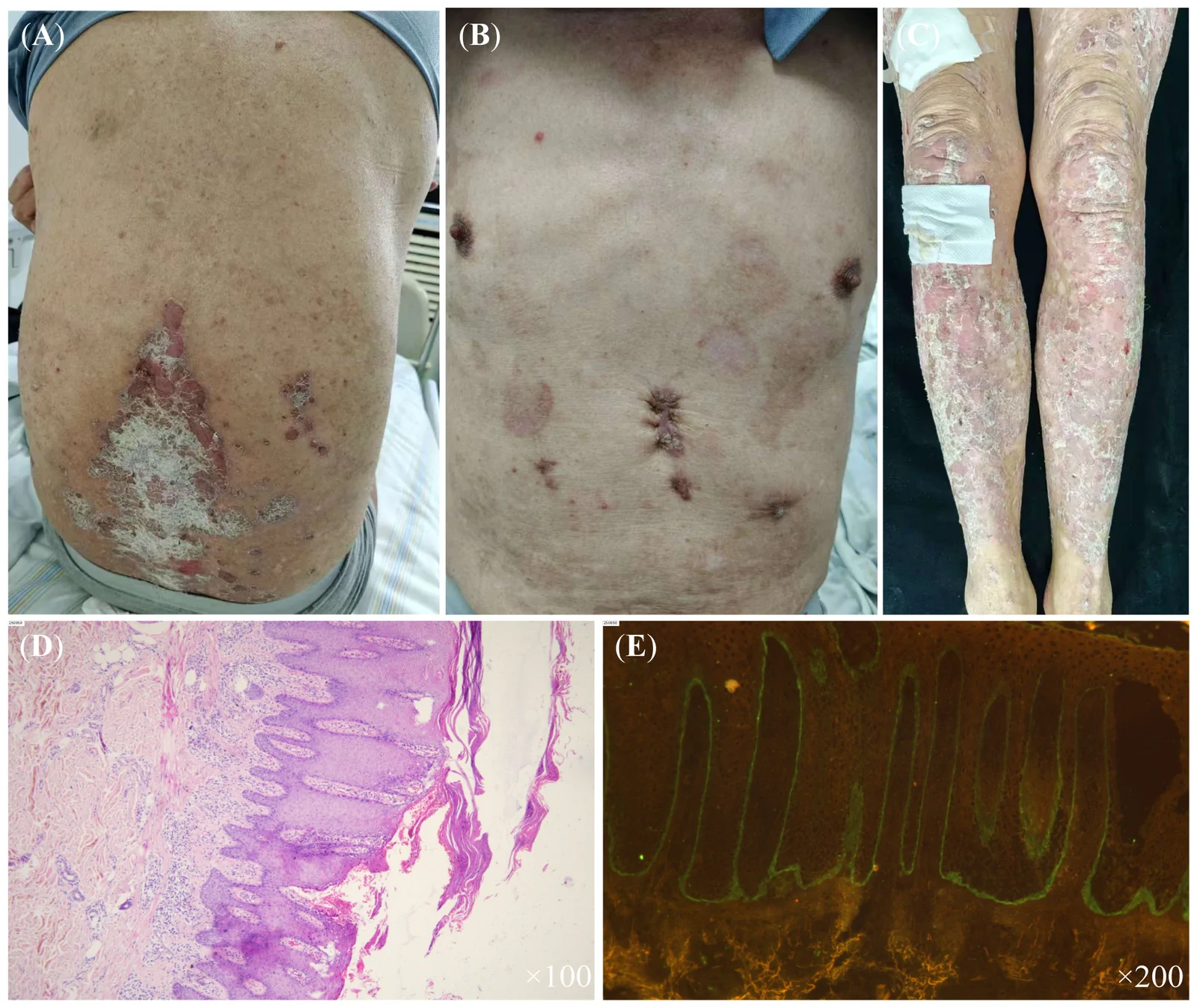
Clinical, histopathological, and immunopathological findings during follow-up in June 2025. **(A-C)** Extensive erythematous scaly plaques and erosions remained on the trunk and extremities after approximately 5 months of ustekinumab treatment, without typical tense vesicles or bullae. **(D)** Histopathological examination showing parakeratosis, neutrophilic aggregates in the stratum corneum, acanthosis, elongation of the rete ridges and dermal papillae, and dilation of superficial dermal vessels, consistent with psoriasiform changes (hematoxylin and eosin staining; original magnification, ×100). **(E)** Direct immunofluorescence showing persistent C3 deposition along the basement membrane zone (original magnification, ×200).

At this time, the peripheral eosinophil percentage was 5.9%, with an absolute count of 0.40 × 10^9^/L. Serum total immunoglobulin E had increased to greater than 1,100 kU/L. Anti-BP180 antibody remained above 400 RU/mL, whereas anti-BP230 antibody had decreased to 10.22 RU/mL. Histopathological examination showed parakeratosis, neutrophilic aggregates in the stratum corneum, acanthosis, and elongation of the dermal papillae, consistent with psoriasiform changes. Direct immunofluorescence continued to show complement component 3 deposition along the basement membrane zone, whereas immunoglobulin G, immunoglobulin A, and immunoglobulin M were negative (**Figure 2**). These findings indicated active plaque psoriasis and clinically quiescent BP, with persistent BP-associated serological and immunopathological abnormalities.

Because psoriasis remained inadequately controlled and the patient declined further biologic treatment, oral deucravacitinib was initiated at a dose of 6 mg once daily after exclusion of relevant contraindications and discussion of the potential benefits and risks. Topical calcipotriol, fluticasone propionate, and tapinarof, together with oral rupatadine, were also prescribed. Following the initiation of deucravacitinib and topical treatment, the psoriatic lesions improved from moderate to mild severity before discharge. No new vesicles or bullae developed, and BP remained clinically quiescent.

The patient continued deucravacitinib until December 2025 and subsequently discontinued the medication on his own. During treatment, psoriasis remained clinically mild, and no recurrent blistering, marked pruritus, erosions, or mucosal involvement was observed. At the latest structured telephone follow-up, approximately 6 months after discontinuation of deucravacitinib, the patient reported that his psoriatic lesions remained mild and stable. He also reported no recurrence of bullae, marked pruritus, erosions, or mucosal involvement. He was receiving topical treatment at another hospital and was not taking systemic therapy. These findings indicated sustained clinical remission of BP throughout the available follow-up period.

## Patient perspective

The patient reported that the sudden onset of widespread blistering and the uncertainty surrounding subsequent systemic treatment caused considerable concern. After receiving several biologic therapies and experiencing recurrent or changing skin lesions, he preferred an oral treatment and declined further biologic therapy. He considered the absence of recurrent blistering reassuring but remained dissatisfied with the control of psoriasis.

Because he did not attend regular follow-up visits, objective post-treatment psoriasis scores and repeat measurements of eosinophils, total immunoglobulin E, BP180 and BP230 antibodies, and direct immunofluorescence findings were unavailable. Therefore, the effect of deucravacitinib on BP-associated immunological activity could not be determined. The complete clinical course, including the major investigations, systemic treatments, and outcomes, is summarized in **Table 1**.

**Table 1:** Key clinical course and treatment timeline.

Time	Psoriasis course	BP findings/status	Treatment and outcome
March 2023	Severe plaque psoriasis (BSA approximately 95%)	No clinical manifestations of BP	Secukinumab 300 mg initiated; marked improvement after two injections
October 2023	Moderate after 10 secukinumab injections	New generalized pruritic tense bullae; eosinophils 16.4% (2.30 × 10^9^/L), total IgE 693 kU/L, anti-BP180 >400 RU/mL, and anti-BP230 344.06 RU/mL; subepidermal eosinophil-rich blister and linear BMZ IgG/C3 on DIF	Secukinumab discontinued; prednisone 30 mg/day, cyclosporine 175 mg/day, minocycline, nicotinamide, and topical corticosteroids initiated; no new bullae after 3 days
October 2023–April 2024	Mild to moderate	Clinically controlled; eosinophil count normalized	Prednisone reduced by 5 mg every 4 weeks; prednisone and cyclosporine discontinued in April 2024
May–September 2024	Transient improvement	No clinical recurrence	Tildrakizumab discontinued after eczematous lesions developed
January–June 2025	Moderate; inadequately controlled	Clinically quiescent	Ustekinumab administered for approximately 5 months; inadequate psoriasis response
June–December 2025	Improved from moderate to mild	No bullae; in June, total IgE >1,100 kU/L, anti-BP180 >400 RU/mL, anti-BP230 10.22 RU/mL, and persistent linear BMZ C3 on DIF; no clinical recurrence during treatment	Deucravacitinib 6 mg/day plus topical treatment for approximately 6 months
Latest follow-up*	Mild and stable by patient report	No reported clinical recurrence	Topical treatment only

*Approximately 6 months after deucravacitinib discontinuation. BMZ, basement membrane zone; BP, bullous pemphigoid; BSA, body surface area; DIF, direct immunofluorescence; IgE, immunoglobulin E.

## Discussion

Psoriasis and bullous pemphigoid (BP) are immunologically distinct diseases, but epidemiological studies have demonstrated a bidirectional association between them (1, 2). Long-standing psoriatic inflammation, repeated epidermal injury, and previous ultraviolet B phototherapy may disrupt the dermoepidermal junction, increase the exposure or alter the antigenicity of BP180 and BP230, and promote epitope spreading and loss of immune tolerance (3, 4). This process may establish susceptibility to BP before blistering becomes clinically apparent.

The immunological relationship between psoriasis and BP extends beyond this structural susceptibility. Psoriasis is predominantly driven by the IL-23/Th17 axis, whereas BP is characterized mainly by type 2 inflammation, eosinophil activation, IgE and IgG autoantibody production, and complement activation. BP nevertheless exhibits a mixed type 2/type 17 cytokine environment in a subset of patients (3, 6, 7). Increased IL-17 expression has been detected in BP lesions and blister fluid. IL-17 can promote neutrophil recruitment and the release of matrix metalloproteinase-9 and neutrophil elastase, thereby contributing to BP180 degradation and dermoepidermal separation (6, 7). Regulatory T-cell dysfunction and keratinocyte- and neutrophil-mediated inflammation also occur in both diseases. Thus, the principal overlap lies in IL-17-related inflammation, impaired immune regulation, and protease-mediated tissue injury, while autoantibodies, complement, eosinophils, and type 2 cytokines remain the dominant drivers of blister formation in BP.

In the present patient, concordant clinical, serological, histopathological, and direct immunofluorescence findings established the diagnosis of BP after 10 injections of secukinumab. The improvement of psoriasis during secukinumab treatment indicates that ongoing psoriatic epidermal injury alone does not adequately explain the timing of BP onset. The patient had experienced severe and extensive psoriasis for more than 20 years and had received conventional treatments, including UVB phototherapy, before biological therapy. Chronic inflammation and repeated injury at the dermoepidermal junction may therefore have established antigen exposure, epitope spreading, and autoreactive T- and B-cell responses before secukinumab was introduced. Clinical improvement of the psoriatic plaques would not necessarily eliminate these pre-existing immune responses.

Secukinumab effectively suppresses IL-17A-driven psoriatic inflammation but does not directly inhibit IL-4, IL-5, IL-13, eosinophil activation, autoantibody production, or complement activation. Selective suppression of the Th17 pathway may alter the relative balance between Th17- and Th2-associated responses and favor type 2, eosinophilic, IgE-related, or autoantibody-mediated inflammation in a susceptible patient. This provides a possible explanation for the development of BP despite improvement of the psoriatic lesions. It also reconciles the apparently opposing observations that IL-17A participates in the inflammatory effector phase of established BP, while IL-17A inhibitors have occasionally been associated with new-onset or relapsing BP (5, 8–10). The effect of IL-17A blockade may therefore depend on disease stage and the patient’s pre-existing immune background. In this case, secukinumab may have shifted subclinical anti-basement membrane zone autoimmunity toward clinically overt BP while continuing to control IL-17-driven psoriasis. The temporal sequence and the improvement of BP after secukinumab withdrawal and BP-directed treatment support its role as a plausible trigger superimposed on the susceptibility created by long-standing psoriasis.

The subsequent clinical course illustrates the challenges of selecting systemic therapy when psoriasis and BP coexist. The previous biological treatments did not all represent primary treatment failure. Secukinumab initially produced marked improvement in psoriasis but was discontinued after BP developed. Tildrakizumab selectively blocks the p19 subunit of IL-23 and therefore mainly suppresses the IL-23/Th17 pathway while preserving IL-12 signaling. It produced transient improvement but was discontinued when eczematous lesions appeared. Ustekinumab targets the p40 subunit shared by IL-12 and IL-23 and consequently affects both IL-12/Th1 and IL-23/Th17 signaling. In this patient, approximately five months of ustekinumab treatment did not provide adequate psoriasis control. Although BP recurrence after ustekinumab re-exposure has been reported (11), no clinical recurrence occurred during ustekinumab treatment in our patient. These different outcomes indicate that drugs acting at separate points within related cytokine pathways may produce distinct clinical effects in an individual patient.

Another important feature was the dissociation between clinical remission and persistent BP-associated immunological abnormalities. In June 2025, the patient had no typical tense bullae and was considered to be in clinical remission from BP. Anti-BP180 antibody levels nevertheless remained above 400 RU/mL, total IgE exceeded 1,100 kU/L, and direct immunofluorescence continued to demonstrate linear C3 deposition along the basement membrane zone, whereas anti-BP230 antibody levels had markedly decreased. These findings indicate that BP-associated autoimmunity may persist after the disappearance of blisters and that different immunological markers may decline at different rates. Persistent serological or immunopathological abnormalities do not by themselves define a clinical relapse, but they support continued surveillance and interpretation alongside pruritus, new lesions, eosinophil counts, and longitudinal clinical findings.

Because the patient declined further biological therapy and preferred an oral treatment, deucravacitinib was selected for persistent moderate psoriasis. Close observation was warranted because new-onset BP shortly after deucravacitinib initiation has recently been reported (12). Deucravacitinib selectively and allosterically inhibits the regulatory domain of TYK2, thereby suppressing intracellular signaling downstream of IL-23, IL-12, and type I interferons (13). Its signaling profile differs from that of both tildrakizumab and ustekinumab. Tildrakizumab selectively blocks IL-23, and ustekinumab blocks IL-12 and IL-23 through their shared extracellular p40 subunit. Deucravacitinib modulates the intracellular signaling of all three TYK2-dependent cytokine groups and may therefore simultaneously affect IL-23/Th17 activity, IL-12/Th1 responses, type I interferon signaling, and downstream keratinocyte activation. This combined effect, together with adequate treatment duration, oral adherence, and concomitant topical therapy, may explain the favorable psoriasis response observed in this patient despite the limited responses to preceding biologics.

The potential effect of TYK2 inhibition on BP is more complex. By reducing IL-23-driven Th17 activation, deucravacitinib may indirectly decrease neutrophil and eosinophil recruitment, protease release, and basement membrane zone injury associated with the type 17 component of BP. It does not directly block IL-4, IL-5, or IL-13, so any effect on the dominant type 2 and eosinophilic components of BP would be indirect. Lu et al. reported BP arising during deucravacitinib treatment (12), whereas a more recent case described sustained control of both psoriasis and BP during deucravacitinib therapy (14). In our patient, BP was already clinically quiescent before deucravacitinib was initiated, and no recurrent blistering, marked pruritus, erosions, or mucosal involvement was observed during approximately six months of treatment or during the available follow-up after discontinuation. Taken together, these contrasting observations suggest that the effect of TYK2 inhibition on BP may vary according to baseline BP activity, the relative contribution of type 2 and type 17 inflammation, previous treatments, and concomitant therapy. Patients with current or previous BP should therefore be monitored for new pruritus, vesicles, tense bullae, erosions, and mucosal lesions when deucravacitinib is introduced.

The principal limitations of this report are the absence of baseline anti-BP180 and anti-BP230 antibody measurements before secukinumab treatment, the absence of drug rechallenge, and incomplete longitudinal PASI and BPDAI assessments. In addition, post-deucravacitinib BPDAI scores, BP-related serological markers, eosinophil counts, total IgE levels, and repeat direct immunofluorescence findings were unavailable; thus, follow-up after deucravacitinib documents clinical non-recurrence rather than changes in BP-associated immune activity. Overall, this case supports a layered pathogenic model in which long-standing psoriasis creates susceptibility through chronic inflammation and basement membrane zone injury, while secukinumab-associated immune rebalancing may promote the transition from subclinical autoimmunity to clinically overt BP. It also demonstrates that clinical remission may precede immunological remission and that treatment selection should account for the distinct but interacting immune pathways of psoriasis and BP.

## Conclusion

In this patient, BP developed during secukinumab treatment despite improvement of long-standing psoriasis. The clinical course supports a possible mechanism in which chronic psoriatic inflammation establishes susceptibility to BP, while IL-17A blockade alters the balance between Th17- and type 2-associated immune responses and promotes clinically overt disease. Persistent immunological abnormalities after blister resolution highlight the need for continued clinical and immunological monitoring when switching targeted therapies.

## Data Availability

The original contributions presented in the study are included in the article. Additional patient-level data are not publicly available because of patient privacy and confidentiality. Further inquiries can be directed to the corresponding authors and will be considered in accordance with the patient’s informed consent and institutional requirements.
